# Supplementary material for: Autoantibody against Tumor-Associated Antigens as Diagnostic Biomarkers in Hispanic Patients with Hepatocellular Carcinoma
Source: Cells. 2022 Oct 14;11(20):3227. doi: 10.3390/cells11203227 (PMC9600682; doi:10.3390/cells11203227)
Supplement: Supplementary file 1 [file cells-11-03227-s001.zip › cells-1922486-supplementary.pdf]

Supplementary Figure S1.

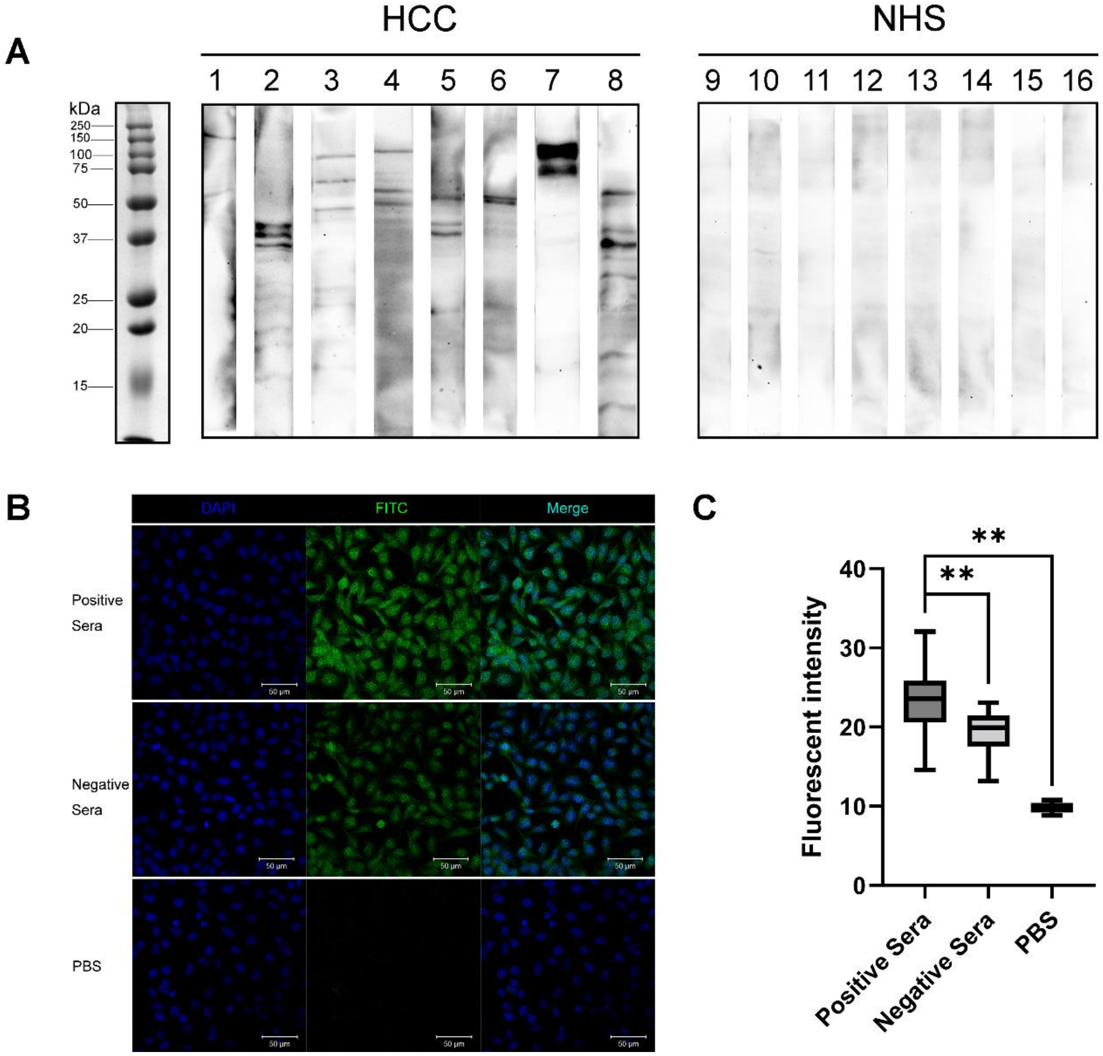

"Positive sera" were screened by western blotting and indirect immunofluorescence analysis. The proteome of HepG2 cell lysates was incubated with 24 Hispanic HCC sera and 40 normal healthy sera (NHS) samples. Samples with prominent immune-reactive bands in HCC were deemed "positive sera" and samples without blotting bands in NHS were "negative sera". Both groups were verified with a separate indirect immunofluorescence assay. (A) Western blotting of representative serum samples with HepG2 cell line. Lanes 1-8 are Hispanic HCC sera with strong immune-reactive bands, and lanes 9-16 are NHS with weak or no immune-reactive bands; (B) Representative immunofluorescent images of "positive sera" from HCC, "negative sera" from NHS, and PBS; (C) Different immunofluorescent intensity was found between "positive sera" group to "negative sera" and PBS groups. PBS was used as blank control. **: $p < 0.01$.

Supplementary Figure S2.

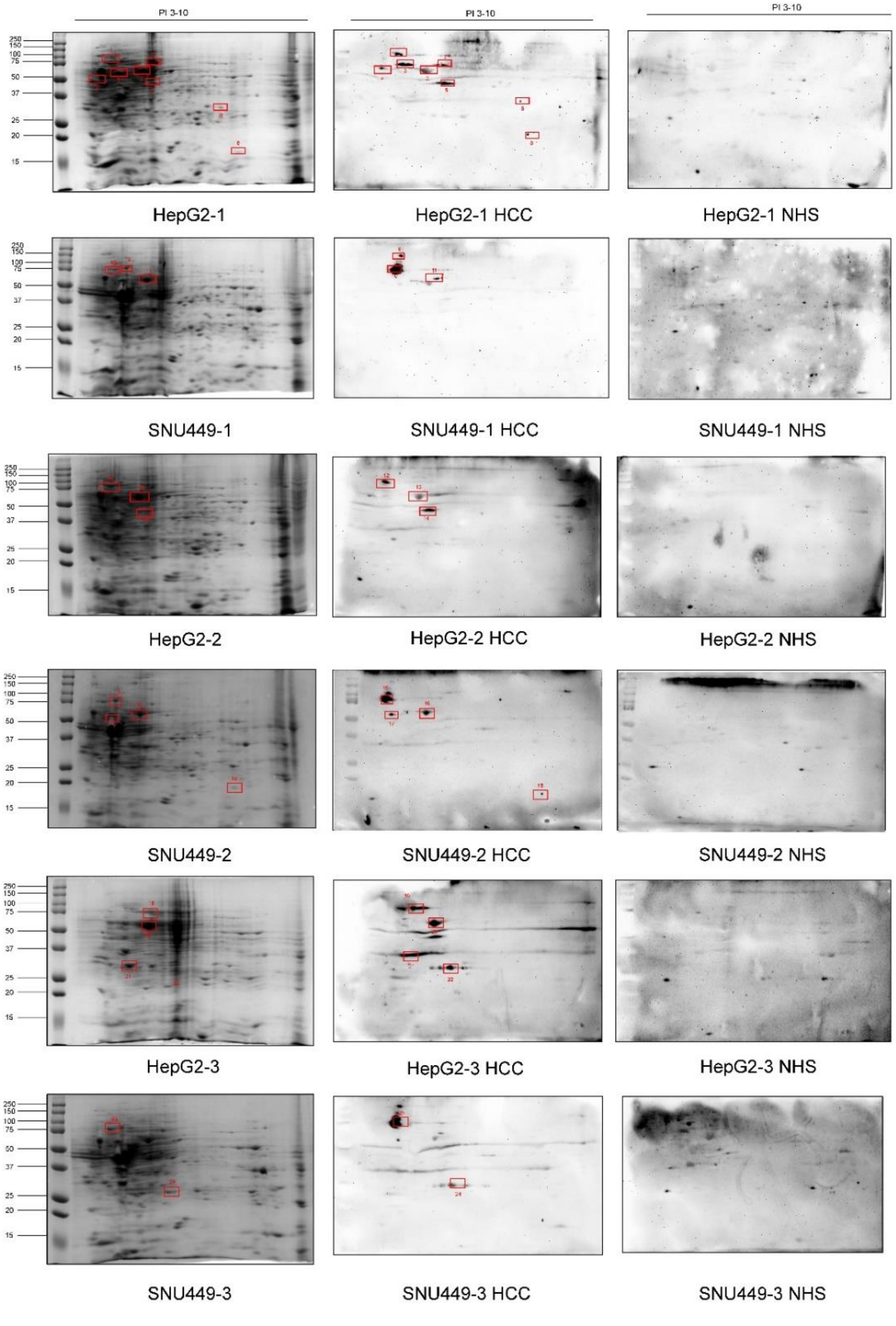

SERPA was used to identify 24 differentially expressed protein spots. HepG2 and SNU449 cell lysates provided the proteome of HCC. SDS-PAGE gels were stained with Coomassie blue, or incubated with "positive sera" pool, or incubated with "negative sera" pool, respectively. Experiments were repeated 3 times. Proteins highly expressed in HCC while not expressed in NHS were screened out and excised for further mass spectrometry analysis. 1~24: Differentially expressed protein spots.
